# Twelve-Week Mediterranean Diet Intervention Increases Citrus Bioflavonoid Levels and Reduces Inflammation in People with Type 2 Diabetes Mellitus

**DOI:** 10.3390/nu13041133

**Published:** 2021-03-30

**Authors:** Hayder A. Al-Aubaidy, Aanchal Dayan, Myrna A. Deseo, Catherine Itsiopoulos, Dina Jamil, Najah R. Hadi, Colleen J. Thomas

**Affiliations:** 1 Department of Physiology, Anatomy and Microbiology, School of Life Sciences, La Trobe University, Melbourne 3086, Australia; aanchal.dayan97@hotmail.com (A.D.); d.jamil@latrobe.edu.au (D.J.); colleen.thomas@latrobe.edu.au (C.J.T.); 2 ARC Research Hub for Medicinal Agriculture, La Trobe Institute for Agriculture and Food, Department of Animal, Plant and Soil Sciences, School of Life Sciences, La Trobe University, Melbourne 3086, Australia; M.Deseo@latrobe.edu.au; 3 School of Allied Health, Human Services and Sport, La Trobe University, Melbourne 3086, Australia; c.itsiopoulos@latrobe.edu.au; 4 Department of Pharmacology and Therapeutics, Faculty of Medicine, University of Kufa, Al-Najaf 0054, Iraq; drnajahhadi@yahoo.com

**Keywords:** type 2 diabetes mellitus, dipeptidyl peptidase-4, citrus bioflavonoids, oxidative stress, inflammation

## Abstract

The benefits of a Mediterranean Diet (MedDiet) in the management of diabetes have been reported, but the contribution of polyphenol-rich citrus fruit has not been studied widely. Here, we report the sub-study findings of a previously conducted MedDiet intervention clinical trial in patients with type 2 diabetes mellitus (T2DM), where we aimed to measure the diet intervention effects on plasma citrus bioflavonoids levels and biomarkers of inflammation and oxidative stress. We analysed plasma samples from 19 (of original 27) participants with T2DM who were randomly assigned to consume the MedDiet intervention or their usual diet for 12 weeks and then crossed over to the alternate diet. Compared with baseline, MedDiet significantly increased levels of the citrus bioflavonoids naringin, hesperitin and hesperidin (by 60%, 58% and 39%, respectively, *p* < 0.05) and reduced plasma levels of the pro-inflammatory cytokine IL-6 (by 49%, *p* = 0.016). Oxidative stress marker 8-hydroxy-2′-deoxyguanosine (8-OHdG) decreased by 32.4% (*p* = 0.128). Usual diet did not induce these beneficial changes. The reduced inflammatory profile of T2DM participants may, in part, be attributed to the anti-inflammatory actions of citrus bioflavonoids. Together with indications of improved oxidative stress, these findings add to the scientific evidence base for beneficial consumption of citrus fruit in the MedDiet pattern.

## 1. Introduction

Type 2 diabetes mellitus (T2DM) is a worldwide epidemic with increasing prevalence. It develops as a result of hereditary and/or environmental factors, in conjunction with sedentary lifestyle and rising levels of obesity [1]. T2DM is characterised by persistent hyperglycaemia, insulin resistance and hyperinsulinemia, and it is commonly defined as a form of metabolic syndrome (MetS). Treatment options vary between pharmaceutical intervention (such as the use of oral hypoglycaemics) and lifestyle change, with a reduction in weight via exercise and diet being most preferable. The three main pathophysiological drivers contributing to hyperglycaemia in T2DM are insulin resistance, oxidative stress and inflammation [2].

Oxidative stress is commonly observed in MetS and T2DM, as elevated glucose levels stimulate the production of reactive oxygen species (ROS) such as hydrogen peroxide and free radicals [3,4]. Oxidative stress contributes to cellular injury such as β-cell dysfunction, insulin resistance and impaired glucose tolerance, all of which contribute to the worsening of pancreatic islet β-cell function, causing a deficiency in insulin release [5]. Atherosclerosis in T2DM results from insulin resistance, which increases ROS production from free fatty acids, leading to increased blood levels of total cholesterol and Low-density lipoproteins (LDL; the atherogenic cholesterol) [6,7]. Consequently, persistent expression of pro-inflammatory proteins such as interleukin-6 (IL-6), interleukin-1β and tumour necrosis factor-alpha (TNF-⍺) may occur, even when glycaemic levels are controlled [8]. Exercise may counter excessive levels of oxidative stress by increasing antioxidant defences within the body, improving metabolic and inflammatory outcomes [9].

The oral hypoglycaemic drugs are used primarily for glycaemic control, particularly in T2DM progression where further loss of insulin production and β-cell function is observed [10]. Metformin, belonging to the biguanide class of drugs, is an insulin sensitiser which lowers hepatic glucose production and promotes glucose uptake in peripheral tissues and is currently the first treatment choice for those with T2DM [11,12]. Dipeptidyl peptidase-IV (DPP-4) inhibitors, also known as gliptins, are a commonly used second line treatment to control blood glucose levels by inhibiting the DPP-4 enzyme, which ultimately results in increased insulin and decreased glucagon secretion [13].

The Mediterranean Diet (MedDiet) is a plant (vegetable and fruit) rich diet, commonly recommended in patients with chronic disease [14]. The MedDiet consists of foods rich in polyphenols, which possess high antioxidant and anti-inflammatory properties (e.g., fruits, vegetables and extra virgin olive oil) [15,16]. With respect to diabetes, polyphenols are beneficial as they protect pancreatic β-cells against glucose toxicity and inhibit protein enzymes such as α-amylases, which increase starch digestion [17]. At present, polyphenols have been proposed as potential natural diabetic medications that interact with many mechanisms, such as restoration of pancreatic β-cell integrity, prompting tissues to recognise and absorb glucose appropriately and, hence, improving insulin sensitivity [16,18]. We previously reported that HbA1c, a marker of glycaemic index, fell significantly from 7.1% to 6.8% in participants with well-controlled T2DM after 12 weeks on the MedDiet [19].

Citrus bioflavonoids (naringenin, hesperidin and eriocitrin) are a group of natural plant chemicals characterised by varying phenolic structures [20]. They are commonly found in fruits, vegetables and beverages such as wine and tea and known for their antioxidant activities [21]. The predominant citrus fruits are oranges, lemons, limes and grapefruit, and these are featured in the MedDiet pattern. A growing body of evidence indicates that citrus fruits, in particular orange and lemon citrus which are abundant in polyphenols, are powerful antioxidants and can also reduce lipid metabolism and improve glucose levels [22]. However, there are limited published data relating the citrus bioflavonoids to cardiometabolic disease [21,22]. In a zebrafish overfeeding model, eriocitrin improved dyslipidaemia and decreased lipid droplets in the liver, an action attributed to activating mitochondrial biogenesis [23]. Moreover, in HepG2 human hepatocarcinoma cells, eriocitrin induced gene expression related to mitochondrial function and reduced lipid accumulation [23]. Hesperidin and naringin are known to reduce blood glucose by altering the original mechanism of glucose-regulating enzymes [24]. In experimental diabetic rats induced with streptozotocin, oral administration of hesperidin showed positive effects on insulin, triglycerides and LDL-C, suggesting potential benefits for T2DM [25]. We recently made the striking discovery that, in addition to acting as antioxidants, citrus bioflavonoids are powerful inhibitors of DPP-4 activity, with in silico and in vitro potency and efficacy profiles comparable to those of gliptins [20]. Whether such activity can be demonstrated in vivo has not been described before. Hence, in this study, we aimed to determine the effects of a MedDiet intervention on plasma citrus bioflavonoid levels, biomarkers of inflammation and oxidative stress and DPP4 activity in patients with T2DM.

## 2. Materials and Methods

### 2.1. Study Design

We analysed previously collected and stored samples from a MedDiet intervention trial conducted by one of our chief investigators (Professor C. Itsiopoulos) [19]. This was a randomised, parallel and crossover intervention study, conducted over a 24-week period in Australian-born people not previously exposed to a traditional Mediterranean type of diet. In brief, 31 participants with well-controlled T2DM from Anglo Celtic backgrounds, aged 47–55 years, were recruited and commenced the trial. Four subjects withdrew due to complicating illnesses of an inability to complete the dietary intervention. Thus, 27 participants successfully completed the study. The study was approved by the Human Research Ethics Committees of both Deakin University and Monash University (Victoria, Australia) and the work was funded by grants from the National Health and Medical Research Council (Application No. 974098) and Diabetes Australia. Of the 27 study completers, 14 participants were randomised to commence on the MedDiet intervention and 13 participants were randomised to commence on their usual (habitual) diet for 12 weeks, and then they crossed over to the alternate diet for the following 12 weeks. The study design did not include a washout period [19].

The intervention MedDiet diet was based on a reconstruction of the traditional Cretan Mediterranean diet [19]. Some key aspects about delivery of the intervention included that, participants were provided with a booklet guide to the Cretan diet (see Supplementary Materials). Approximately 70% of the intervention diet foods were provided to participants. A rolling 2-week cycle menu during the 12-week intervention provided 14 different traditional cooked meal options for lunch and dinner, respectively (including cooked wild edible greens dressed with extra virgin olive oil and lemon juice). Intervention MedDiet meals were prepared in bulk, using traditional methods, packaged into individual portion sizes and stored at −20 °C until required. Additionally, salads accompanied the lunch and dinner menus; some salads were provided (such as cooked leafy green vegetables), whereas some salads needed to be prepared by the participants themselves. Other foods included traditional wholegrain bread, olives, dried fruit, nuts, Greek coffee and herbal tea and extra virgin olive oil as the main dietary fat [19]. The booklet recommended food choices for breakfast, which the participants were required to prepare themselves and consume. Participants were also consulted individually by an accredited practicing dietitian (APD) and advised to consume 3 servings of fruit per day, including citrus fruits high in bioflavonoids and polyphenol content [19]. Participants were advised to consume the intervention diet ad libitum, and they were asked to record actual consumption (food diary) and discard any uneaten food. Adherence to the intervention diet was assessed via a self-completed 7-day diet record (in household measures) at baseline and at the end of the 12-week study period. Relevant plasma biomarkers, including monounsaturated fatty acids and serum carotenoids lutein and lycopene, were also measured [19].

### 2.2. Sample Collection

As part of the trial, blood samples were collected from participants at timed intervals (baseline, post-MedDiet and post-usual diet) and stored at −80 °C [19]. Out of the total 27 participants in the original study, there was enough remaining stored plasma from 19 participants from three key protocol time-points (baseline, end-Med diet intervention and end-usual diet) to undertake the present subset study analysis. In addition, some of the prior measured and relevant variables from the 19 participants in the original study were extracted, collated and are presented herein to inform our new measurements. Glycosylated haemoglobin (HbA1c) was measured in ethylenediaminetetraacetic acid (EDTA)-treated whole blood using an automatic high-performance liquid chromatography (HPLC) analyser (DIAMAT TM Analyzer, Bio-Rad, Hercules, CA, USA; CV 4.1e5.4%) [19]. Total cholesterol, HDL cholesterol, triglyceride and plasma glucose concentrations were analysed on an automatic analyser (Hitachi model 705, Tokyo, Japan) using enzymatic kits (Boehringer Mannheim GmbH Diagnostic, Mannheim, Germany) [20]. Body mass index (BMI) was calculated using the formula BMI = kg/m^2^. LDL cholesterol was calculated via the Friedewald formula and plasma insulin was measured via radioimmunoassay using specific antibodies (Linco Research Inc., Irvine, CA, USA) as per the manufacturer’s instructions [19].

### 2.3. Biomarker Measurements in This Study

Plasma levels of pro-inflammatory cytokine interleukin-6 (IL-6) were measured using a human Elisa Kit (Abcam, ab46027, Boston, MA, USA). The detection sensitivity was 2 pg/mL, and samples were measured in duplicate as per the manufacturer’s instructions. 8-hydroxy 2 deoxyguanosine (8-OHdG) is one of the predominant markers of DNA oxidative damage and has therefore been widely used as a biomarker for oxidative stress [26]. 8-OHdG levels were measured using an ELISA kit (Abcam, ab201734, Boston, MA, USA). The detection sensitivity was 0.59 ng/mL, and samples were measured in duplicate. The activity of dipeptidyl peptidase-IV (DPP4) was assessed using a DPP-4 activity assay kit (Abcam, ab204722, Boston, MA, USA). The detection sensitivity was 3 µIU/µL, and samples were measured in triplicate.

### 2.4. Measurement of Citrus Bioflavonoids in Plasma

Ultra-performance liquid chromatography coupled with triple quadrupole mass spectrometer (UPLC-QqQ MS) was used to identify and quantitate plasma citrus bioflavonoid levels from the participant samples. The first step was to determine and identify the molecular mass and molecular structures of the individual citrus bioflavonoid forms. In particular, they exist as consumed (glycoside) forms (naringin, hesperidin and eriocitrin) or metabolised (aglycone) forms (naringenin, hesperitin and eriodictyol) (Table 1). The two forms were measured to capture all the citrus bioflavonoid content in the plasma. In addition, rutin, a commonly researched flavonoid, was included as a reference standard, but, due to the sample limitation, we only recorded the data for nine participants with full data across the three study groups.

The chromatography was conducted utilising a Vanquish UPLC system with quaternary pump, temperature-controlled column compartment and autosampler (Thermo Fisher Scientific Inc., Waltham, MA, USA) on a Waters Acquity^®^ UPLC BEH C18 1.7 μm column (2.1 × 100 mm) (Waters Corporation, Milford, MA, USA). The mobile phase comprised of 0.1% (*v/v*) formic acid (solvent A) and 0.1% acetonitrile (solvent B) both Optima^®^ LCMS grade and purchased from Thermo Fisher Scientific Australia (Scoresby, VIC, Australia). Elution comprised of an initial value of 10% B to a gradient of 40% solvent B over 7 min, held at 40% B for 1 min, then to 100% B over 1 min and held at 100% B for additional 2.5 min before returning to the starting gradient of 10% B within 0.5 min and equilibrated column for 3 min. Total run time including column equilibration was 15 min and column temperature was 45 °C. The flow rate was 0.35 mL/min using 3 µL injection volumes. The UPLC was coupled to a Thermo Fisher Vantage TSQ mass spectrometer (Thermo Fisher Scientific Inc., Waltham, MA, USA). Detection was made using multiple reaction monitoring (MRM) in negative ionisation mode using a heated electrospray ion source. Capillary temperature was 325 °C, spray voltage was 3.5 kV, vaporiser temperature was 300 °C and sheath gas and auxiliary gas pressures were 40 and 10 arbitrary units, respectively. The collision energies used for each compound’s MRM transition are summarised in Table 1. Data analysis was performed using Xcalibur 2.2 and LCquan 2.1 software (Thermo Fisher Scientific Inc., Waltham, MA, USA). Peak identity was determined by comparison of retention time with reference standards and integration of extracted ion peaks. The levels of eriocitrin and eriodictyol in the samples could not be detected using the current methodology, hence we do not report them in the Results Section 3.2.

### 2.5. Statistical Analysis

All values are expressed as mean ± SEM. Graphs were prepared using SigmaPlot software (version 14, San Jose, CA, USA). Data analysis was performed using the statistical software program SigmaStat (version 14, San Jose, CA, USA). One-way repeated measures analysis of variance (one-way RM ANOVA) was used to determine effect of MedDiet versus usual diet, compared to baseline, for the following indices: haemodynamic; glycaemic status, lipid profile, antioxidant and ant-inflammatory markers and citrus bioflavonoids. Structural equation modelling was used to analyse the potential associations between changes in the plasma levels of DPP-4, BGL and HbA1c following the MedDiet intervention with the changes in the plasma levels of citrus bioflavonoids (rutin, hesperidin, hesperidin, naringin and naringenin). Pearson correlation tests were performed to determine the relationship between the levels of citrus bioflavonoids and IL-6 or 8-OHdG. For all data analyses, significance was accepted if *p* < 0.05.

## 3. Results

### 3.1. Baseline Characteristics of the Participants

The baseline characteristics of the total cohort of 19 participants, and based on sex, are summarised in Table 2. Briefly, the mean age of the participants was 57 ± 1 years, and they were mostly males (58%). With regards to measures of anthropometry, the mean BMI of the participants was 31.54 ± 1.19 kg/m^2^ and waist circumference was 104.12 ± 3.14 cm [1]. The participants had well-controlled diabetes, indicated by the long-term indicator of blood glucose, HbA1c. This is not surprising since 2/3 of the cohort were taking prescribed oral hypoglycaemic mediations and the other 1/3 of participants were on diet therapy only. The participants were normotensive (with normal heart rate) but notably, 50% were taking anti-hypertensive medications. Moreover, participants of this study had higher average levels of total cholesterol and triglycerides (5.38 ± 0.26 and 2.36 ± 0.27 mmol/L, respectively) at baseline, and 25% of participants were taking statins. There were no significant differences in any baseline parameter between males and females, except the females had a higher mean resting heart rate (by ~15 bpm, *p* < 0.05, (Table 2).

### 3.2. Effect of MedDiet on the Levels of Citrus Bioflavonoids

**Figure 1:**
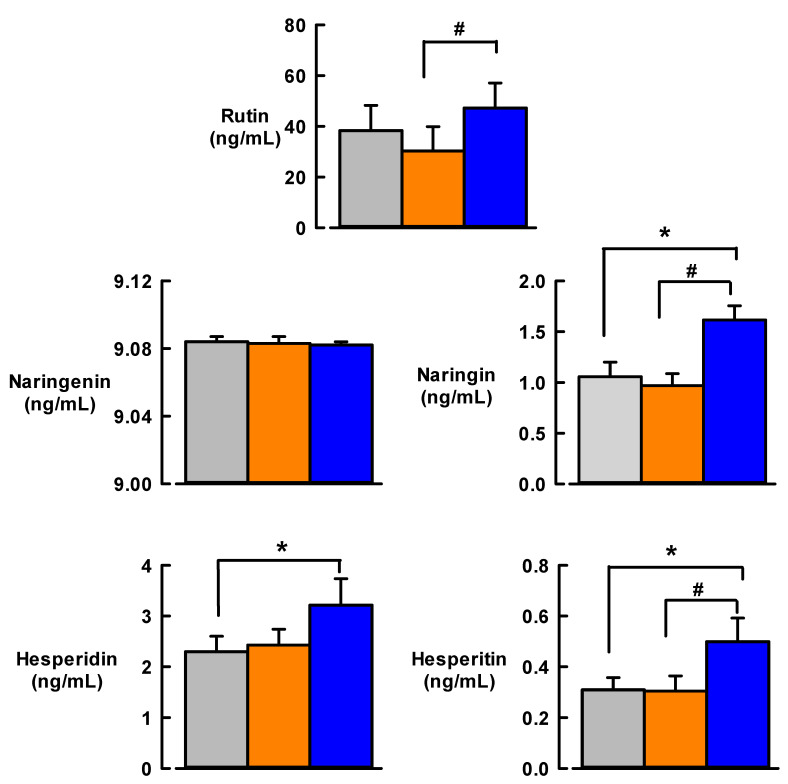
Effect of 12-weeks Mediterranean diet intervention (blue bars) or usual diet (orange bars) on citrus bioflavonoid levels compared with baseline levels (grey bars) in participants with Type 2 diabetes. Sample size: *N* = 18 for naringenin, naringin, hesperidin, hesperitin; *N* = 9 for rutin. Data are mean ±SEM. * *p* < 0.05, different from baseline; # *p* < 0.05, different from usual diet.

Figure 1 illustrates the effect of 12 weeks of MedDiet versus usual diet on citrus bioflavonoid levels of rutin, naringenin, naringin, hesperidin and hesperitin in T2DM participant samples. The usual diet did not change the plasma levels of any citrus bioflavonoid compared to baseline. In contrast, the mean plasma levels of naringin, hesperidin and hesperitin were significantly increased in response to MedDiet (by 60%, 39% and 58%, respectively) compared with baseline levels (*p* < 0.05, Table 3). In addition, rutin, naringin and hesperitin levels were significantly higher after MedDiet intervention compared to 12 weeks of usual diet (*p* < 0.05).

### 3.3. Effect of MedDiet on Glycaemic Status

**Figure 2:**
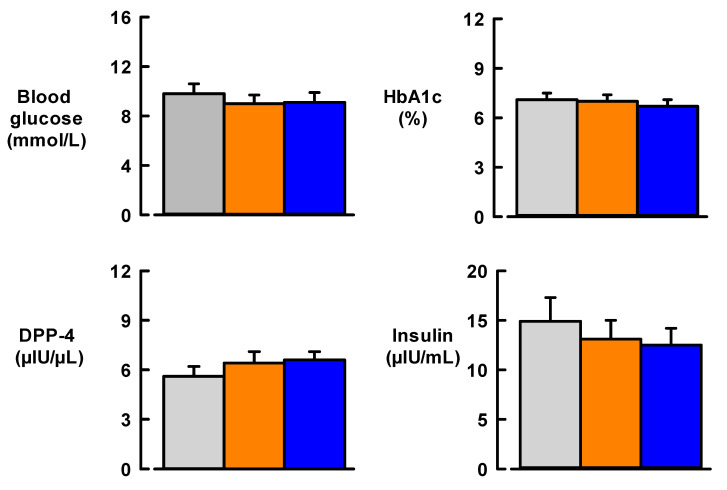
Effect of 12-week Mediterranean diet intervention (blue bars) or usual diet (orange bars) on glycaemic status compared with baseline levels (Grey bars) in participants with type 2 diabetes. Sample size, *N* = 19. Data are mean ± SEM. HbA1c, haemoglobin A1c; DDP4, dipeptidyl-peptidase 4. For insulin (1 µIU/mL = 6.945 pmol/L).

The effect of MedDiet on glycaemic parameters is illustrated in Figure 2. At baseline, the mean blood glucose of participants was 9.8 ± 0.8 mmol/L and this did not significantly change after 12 weeks of MedDiet or usual diet. For all participants, mean HbA1c, the longer-term marker of glucose in the blood, was 7.0 ± 0.4% at baseline and did not change after MedDiet or usual diet (Figure 2). There was a small but non-significant fall (by 2.38 µIU/mL) in insulin levels after MedDiet compared to baseline (*p* = 0.504). Insulin levels also remained unchanged compared with baseline after 12 weeks of usual diet (Figure 2). Finally, there were small but non-significant increases in DPP-4 (*p* = 0.128), an enzyme which inactivates the hormone incretin that helps the body produce insulin, after usual diet and MedDiet (Figure 2).

Structural equation modelling revealed potential estimated correlations between changes in plasma markers of glycaemic status and changes in plasma levels of citrus bioflavonoids following MedDiet intervention. Changes in plasma levels of all citrus bioflavonoids were predicted to correlate to changed BGL levels after MedDiet. In addition, changes in plasma levels of all citrus bioflavonoids except hesperidin were predicted to correlate to changed HbA1 levels after MedDiet. In contrast, only changed hesperitin levels were predicted to correlate with changed DPP-4 levels after MedDiet (Table 2).

### 3.4. Effect of MedDiet on Markers of Inflammation and Oxidative DNA Stress

**Figure 3:**
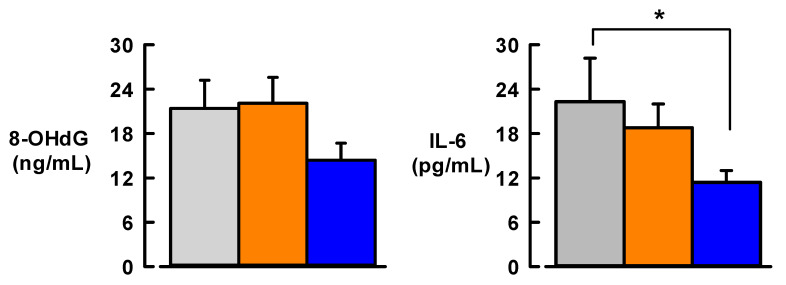
Effect of 12-week Mediterranean diet intervention (blue bars) or usual diet (orange bars) on plasma levels of pro-inflammatory cytokine interleukin-6 (IL-6) and oxidative stress marker 8-hydroxy-2′-deoxyguanosine (8-OHdG) compared with baseline levels (Grey bars) in participants with type 2 diabetes. Sample size, N = 19. Data are mean ± SEM. * *p* < 0.05, different from baseline.

Levels of the pro-inflammatory cytokine IL-6 were significantly decreased (by 49%, *p* < 0.05) after 12 weeks of MedDiet (Figure 3). In contrast, baseline levels of IL-6 did not change following 12 weeks of usual diet (Figure 3). After 12 weeks of MedDiet, there was a small but non-significant fall in plasma levels of the oxidative stress marker 8-OHdG (by ~7 ng/mL from baseline, *p* = 0.128) (Figure 3). The usual diet did not change 8-OHdG levels compared with baseline.

Further analysis revealed that, following MedDiet intervention, IL-6 levels were negatively correlated with naringin (*p* = 0.049) and 8-OHdG levels were negatively correlated with hesperidin (*p* = 0.01). No other citrus bioflavonoid levels following the MedDiet intervention correlated with IL-6 and 8-OHdG.

### 3.5. Effect of MedDiet on Anthropometric Measurements and Lipid Profile

The participants had a significant reduction in BMI (by 0.85 ± 0.1 kg/m^2^, *p* < 0.001) after 12-week MedDiet intervention, compared with baseline. There was no change in BMI after usual diet. After MedDiet, the participants also exhibited significantly reduced body weight (by 2.4 ± 0.1 kg, *p* < 0.001) and waist circumference (by 3.1 ± 0.2 cm, *p* = 0.009) compared with baseline. There was a small but significant (*p* = 0.04) reduction in LDL cholesterol levels of the participants after 12 weeks of usual diet or MedDiet (by 0.25 and 0.24 mmol/L, respectively) compared to baseline. Total cholesterol levels were 5.4 ± 0.3 mmol/L at baseline and did not change significantly after MedDiet or usual diet. Similarly, mean triglyceride levels in the participants did not change significantly after MedDiet or usual diet, compared with baseline. The mean HDL cholesterol was 1.0 ± 0.1 mmol/L at baseline, and these levels did not change significantly after 12 weeks of MedDiet or usual diet.

## 4. Discussion

The key finding from this study was that plasma levels of citrus bioflavonoids (naringin, hesperidin and hesperitin) increased significantly from baseline following 12-week Mediterranean diet intervention in subjects with T2DM. Additionally, and in contrast to usual diet, MedDiet significantly reduced plasma IL-6 and tended to reduce 8-OHdG levels in participants, indicative of improved inflammatory and oxidative stress status. To the best of our knowledge, this is the first study to develop and optimise a protocol for measuring citrus bioflavonoids levels in human plasma. In addition, this is the first study to report levels of citrus bioflavonoids in patients with T2DM following MedDiet consumption.

The glycaemic status of participants did not show a significant improvement after MedDiet, as reflected by non-significant changes in BGL, HbA1c, insulin and DPP-4 enzymatic activity. These results were not surprising due to the small sample size in the present subset study (19 participants). However, in the original study which had 27 participants, there was a significant improvement in the levels of HbA1c in response to 12-week MedDiet intervention in T2DM (BGL and serum insulin did not change) [19]. In addition, the structural equation modelling we performed in this study revealed potential estimated correlations between changes in plasma levels of glycaemic markers (DPP-4, BGL and HbA1c) with changes in plasma levels of citrus bioflavonoids (hesperidin, naringin and rutin) in response to a 12-week MedDiet consumption.

In the parent study [19], adherence to the intervention diet was indicated by changes in plasma biomarkers monounsaturated fatty acids (MUFA), lutein and lycopene. We previously reported that carotenoids have a positive relationship with fruit and vegetable intake [27]. Whilst not measured directly, the contribution of increased citrus fruit intake was reflected in nutrient analyses from the diet records of participants (significantly higher Vitamin C) [19]. The increased levels of citrus bioflavonoids measured in this study can be attributed to the increased consumption by T2DM participants of fruit and vegetables, use of extra virgin olive oil and lemon juice on most salads and the many cooked greens (wild edible greens and vegetables such as broccoli and cauliflower, as well as soups (chicken egg and lemon soup and fish soup) [19]. It is well known that unhealthy diet patterns detrimentally increase the secretion and expression of pro-inflammatory biomarkers and that low grade inflammation promotes chronic CVD [19]. The MedDiet intervention study conducted by Itsiopoulos et al. [19] aimed for improvements in metabolic control and vascular risk in T2DM. Although significant changes in long-term blood glucose control were achieved on the ad libitum MedDiet intervention (reduction of HbA1c from 7.1% (95% CI: 6.5–7.7%) to 6.8% (95% CI: 6.3–7.3%), *p* = 0.012), the study did not demonstrate changes in other CVD risk markers. A larger sample size and longer intervention time may indicate these expected benefits of the MedDiet. It is also worth mentioning that subjects were a relatively low-risk group with clinical inflammation marker, C-reactive protein and blood pressure in the normal range and exhibiting good metabolic control [19]. Citrus bioflavonoids have strong antioxidant effects due to their free radical scavenger properties and actions to prevent cell injury [28].

The current study showed a significant reduction in plasma levels of IL-6 following 12 weeks of MedDiet regimen, something which could not be achieved with the usual diet. The present findings were consistent with a study by Marfella et al. [29] in 115 patients with T2DM and consuming a MedDiet for 52 weeks, which reported a significant 20% reduction in plasma levels of IL-6 in the intervention group (red wine intake) as compared to the control group (no red wine intake). In addition, a non-significant reduction in plasma levels of the oxidative stress marker 8-OHdG was observed in the T2DM participants following 12 weeks of MedDiet. A recent study conducted by Gutierrez Mariscal et al. [30] found that four weeks of MedDiet supplemented with antioxidant coenzyme Q10 significantly reduced 8-OHdG levels in seven subjects. Additional studies have reported reduced urinary levels of 8-OHdG following one-year dietary MedDiet intervention (35 subjects), as well as in peripheral blood leukocytes following three-month MedDiet intervention (30 subjects) [31,32]. It is also known that oxidative DNA damage is present with minor elevations in BGL [1]. Participants in the present study were categorised as overweight or obese at study onset, but BMI levels reduced significantly following the MedDiet intervention. Increased BMI has been linked to increases in DNA damage as a result of oxidative stress, and, as BMI > 30 kg/m^2^ is considered to be “obese”, it is no surprise our participants had elevated levels of oxidative stress at baseline [33,34]. Hence, the trend for reduction of 8-OHdG in response to MedDiet was highly favourable.

A small non-significant rise in DPP-4 activity was observed in T2DM participants after the MedDiet intervention (i.e., indicating the diet pattern tended to reduce insulin release). Whilst at first a surprising finding to us, further investigation of the scientific literature suggests this phenomenon may in fact be a compensatory response to the reduced levels of insulin sensitivity and hyperglycaemia, in an attempt to increase intact incretin hormone concentrations and improve insulin secretion [35,36]. Indeed, another study reported significantly elevated DPP-4 activity in T2DM patients, which may be related to the impaired incretin action [36]. Mannucci et al. [37] reported that DPP-4 activity is related to prevailing glycaemic status of individuals. Significant increases in DPP-4 activity have been associated with high HbA1c levels (>8.5%, well outside the normal range). T2DM patients with decreases in DPP-4 activity had HbA1c levels of 8.0% or less as compared to healthy control subjects [38,39]. Additionally, another study found no change in DPP-4 activity in T2DM [40]. Pala et al. [40] discovered that mild hyperglycaemia did not alter the circulating levels of DPP-4 activity. This may suggest that impairment of GLP-1 incretin hormones response to glucose in early stage T2DM could be due to a reduction of incretin hormone secretion [40]. In addition, this study reported that T2DM patients with well controlled glucose levels (HbA1c ≤ 7%) did not display any differences in DPP-4 activity following oral glucose tolerance test as compared to the T2DM with poor glycaemic control (HbA1C > 7%), inferring that the degree of hyperglycaemia has direct correlation to circulating DPP-4 activity [40].

The significant reduction in HbA1c levels detected in the original study by Itsiopoulos et al. [19] agrees with other MedDiet and T2DM trials [29]. For example, Marfella et al. [29] reported that HbA1c fell significantly in patients with diabetes after 52 weeks. These findings are of clinical relevance and importance, as they point to likely reduced incidence of diabetes-linked complications and overall decreased mortality rate [41]. Insulin levels fell in the original study by Itsiopoulos [19] by ~2 mmol/L after 12 weeks on the MedDiet. Furthermore, the original study showed a trend towards improved insulin sensitivity after MedDiet compared to usual diet (homeostasis model assessment (HOMA) score reduced from the 6.1 (95% CI: 4.4–7.8) to 5.2 (95% CI: 3.9–6.6), *p* = 0.061) [19]. Estruch and colleagues noted improvements in blood glucose levels in T2DM patients, decreased insulin resistance and increased insulin sensitivity, indicating overall improved glycaemic control following three months of MedDiet style diet [42], a similar study to the current one with similar glycaemic parameters and lipid profile [42].

Anthropometric measurements from the present sub-study participants were significantly improved in response to MedDiet intervention, as manifest by significant reduction in BMI associated with similar reduction in body weight and waist circumference. This is very interesting (a trend was observed in the original *n* = 27 study), as the intervention was ad libitum and not energy restricted [19]. Moreover, these beneficial changes agreed well with associated small but significant reductions in LDL cholesterol among the participant group. Major MedDiet interventions such as the landmark PREDIMED study which did not have caloric restriction, have highlighted the benefits of the diet, being the improvements in lipid profile, body composition and reductions in oxidative stress and inflammatory status. Some studies have demonstrated changes in BMI and body fat% following MedDiet trials [43,44]. Prior studies have demonstrated that a reduction in abdominal adiposity and obesity is a common effect of the MedDiet in well controlled clinical trials [45]. Additionally, greater MedDiet adherence results in greater weight loss in obese people than individuals on a low-fat diet [45] and improvement in lipid profile, with reduced total cholesterol and increased HDL cholesterol [46]. Salas- Salvadó and colleagues previously reported that MedDiet increased HDL cholesterol and lowered total cholesterol and triglycerides [47]. It is important to note that, in the present study, 25% of participants were taking statin medications for cholesterol lowering prior to and during the intervention trial, and, due to this particular class of drug and its cholesterol lowering effects, significant changes were not observed for HDLs, total cholesterols and triglycerides.

One of the limitations of this sub-study was that, out of 27 participants in the original trial, there was only sufficient collected and stored plasma from 19 participants to evaluate citrus bioflavonoids, IL-6, 8-OHdG and DPP-4 levels at the three key timepoints in the trial (i.e., baseline, after MedDiet or after usual diet). Further adding to factors that could influence the outcomes of our study, we decided that, for any bioflavonoids, if any participant had a missing study sample (e.g., due to technical difficulties such as below level of assay detection and/or insufficient sample volume), then the entire participant’s dataset for that bioflavonoid would be excluded from the results. Naringin, naringenin, hesperidin and hesperidin levels are reported in eighteen participants, while rutin levels are reported in nine participants only. Nevertheless, this is the first study to measure the circulating levels of citrus bioflavonoids in patients with type 2 diabetes mellitus. The development and optimisation of the assay procedure by our analytical team has provided the basis to continue our program of citrus bioflavonoids research.

## 5. Conclusions

This study found significantly increased plasma levels of citrus bioflavonoids (naringin, hesperitin, hesperidin and rutin), associated with reduced inflammation following a MedDiet intervention in participants with well controlled T2DM. It is also one of the first studies of its kind to investigate the effect of the Mediterranean diet on oxidative stress marker 8-OHdG in plasma samples. Compared to usual diet, 12 weeks of Mediterranean diet caused significant falls in the pro-inflammatory cytokine IL-6. The reduced inflammatory profile of T2DM participants may, in part, be attributed to the anti-inflammatory actions of citrus bioflavonoids. In addition, MedDiet improved the lipid profile of the cohort (decreased LDL). These findings add to the scientific evidence base for beneficial consumption of citrus fruit in the MedDiet pattern.

## Figures and Tables

**Table 1:** Chemical characteristics of the citrus bioflavonoids (eriocitrin, naringenin and hesperidin) compounds in both aglycone forms and glycoside forms compared with rutin flavonoid.

Standard Compounds	Molecular Formula	Molar Mass (g/mol)	Retention Time (min)	Product Ion (m/z)	Collision Energy (V)
Consumed (glycoside) form					
Eriocitrin	C_27_H_32_O_15_	596.50	6.9	287.0	24
Naringin	C_27_H_32_O_14_	580.54	7.5	271.2	31
Hesperidin	C_28_H_34_O_15_	610.57	7.7	301.2	25
Metabolic (aglycone) form					
Eriodictyol	C_15_H_12_O_6_	288.25	8.4	151.1	16
Naringenin	C_15_H_12_O_5_	272.26	9.1	151.2	20
Hesperetin	C_16_H_14_O_6_	302.27	9.4	164.1	26
Rutin	C_27_H_30_O_16_	610.52	6.8	300.2	38

**Table 2:** Descriptive characteristics of the study participants at baseline.

Parameter	Total Cohort (*N* = 19)	Males *N* = 11 (58%)	Females *N* = 8 (42%)
Age (years)	57.5 ± 1.30	58.55 ± 1.80	56.00 ± 2.00
Anthropometric measurements			
Weight (kg)	93.14 ± 3.14	97.00 ± 3.00	87.00 ± 4.00
Waist circumference (cm)	104.12 ± 2.28	105.00 ± 3.00	102.70 ± 3.20
BMI (kg/m^2^)	31.54 ±1.19	30.20 ± 1.00	33.30 ± 2.3
Glycaemic status			
Fasting Glucose (mmol/L)	9.83 ± 0.82	9.40 ± 0.98	10.37 ± 1.40
HbA1c (%)	7.02 ± 0.40	7.00 ± 0.50	7.20 ± 0.50
Insulin (µIU/mL)	14.92 ± 2.38	14.92 ± 2.87	14.91 ± 4.26
DPP-4 (µIU/µL)	5.61 ± 0.59	4.88 ± 0.31	6.60 ± 1.29
Lipid profile			
Total cholesterol (mmol/L)	5.38 ± 0.26	5.10 ± 0.20	5.60 ± 0.06
LDL cholesterol (mmol/L)	3.31 ± 0.20	3.20 ± 0.20	3.40 ± 0.30
HDL cholesterol (mmol/L)	1.00 ± 0.06	0.95 ± 0.07	1.00 ± 0.10
Triglycerides (mmol/L)	2.36 ± 0.27	2.17 ± 0.30	2.60 ± 0.50
Hemodynamic measurements			
Systolic Blood Pressure (mmHg)	136.84 ± 3.18	135.00 ± 3.90	139.30 ± 5.40
Diastolic Blood Pressure (mmHg)	72.95 ± 2.14	73.90 ± 3.10	71.60 ± 2.90
Heart Rate (bpm)	68.63 ± 3.15	62.00 ± 3.80	77.60 ± 3.40 *

Data are Mean ± SEM. BMI, body mass index; HbA1c, haemoglobin-A1c; DPP-4, dipeptidyl peptidase-4 enzyme; LDL, low density lipoprotein; HDL, high density lipoprotein. * *p* < 0.05, different from males.

**Table 3:** Predicted correlations between changes in the plasma levels of DPP-4, BGL and HBA1c and changes in plasma levels of citrus bioflavonoids following MedDiet intervention.

Variables	Estimated Regression Weight * (S.E.)	*p* Value
Changes in the plasma levels of DPP-4 following MedDiet intervention		
Hesperitin	−0.88 (0.31)	0.005
Hesperidin	−0.97 (1.41)	0.48
Naringin	0.12 (0.14)	0.4
Naringenin	21.37 (25.85)	0.4
Rutin	−0.022	0.26
Changes in the plasma levels of BGL following MedDiet intervention		
Hesperitin	524.40 (1.28)	<0.001
Hesperidin	506.90 (4.91)	<0.001
Naringin	−176.70 (0.51)	<0.001
Naringenin	150,922 (90.43)	<0.001
Rutin	60.90 (0.06)	<0.001
Changes in the plasma levels of HbA1c following MedDiet intervention		
Hesperitin	733.80 (97.81)	<0.001
Hesperidin	458.80 (369.70)	0.22
Naringin	−259.90 (38.84)	<0.001
Naringenin	178,300 (6801.56)	<0.001
Rutin	79.70 (5.22)	<0.001

* Estimated regression weights obtained using structural equation modelling. S.E, standard error; DDP-4, dipeptidyl-peptidase 4; BGL, blood glucose levels; HbA1c, haemoglobin A1c.

## Data Availability

The data presented in this study are available on request from the corresponding author. The data are not publicly available due to patients’ privacy.

## Supplementary Materials

The following are available online at https://www.mdpi.com/article/10.3390/nu13041133/s1,Title: booklet guide to the Cretan diet, Data obtained from: Itsiopoulos C. Diabetes and Cardiovascular Disease: The Greek Migrant Paradox. Ph.D. Thesis, University of Melbourne, Melbourne, Australia, 2007.
