# Supplementary material for: Twelve-Week Mediterranean Diet Intervention Increases Citrus Bioflavonoid Levels and Reduces Inflammation in People with Type 2 Diabetes Mellitus
Source: Nutrients. 2021 Mar 30;13(4):1133. doi: 10.3390/nu13041133 (PMC8065815; doi:10.3390/nu13041133)
Supplement: Supplementary file 1 [file nutrients-13-01133-s001.pdf]

Two week cycle
Date:

Cretan Diet

This booklet is a guide to the Cretan diet. The two - week cycle menu provides 14 different options for lunch meals and 14 different options for dinner meals. In addition each lunch and dinner meal is accompanied by a salad, some will be provided, whilst the others will be prepared by you. Remember once you have chosen and consumed that meal, mark it off in the box provided. This will ensure variety and flexibility.

With the measuring spoons that have been provided, you will weigh out approximately the required amount of olive oil that is required for that recipe. Remember to double up on the olive oil if you are cooking for two.

It is imperative that any oil that remains on the plate after a meal should be mopped up with the bread provided.

Foods that have the letter (P) attached to it indicate that the food has been provided. Foods that have the letter (E) attached to it indicates that the food or salad needs to be prepared by you.

On the day you commence the diet we will provide you with a two week supply of food (P). The remaining foods on the two week cycle will be prepared by you (E). You will find all the recipes in the booklet provided

<u>Types of tinned tomatoes that can be used:</u>
Leggos tomato puree
Trident tomato puree
SPC tomato puree
If not one of the above, please specify brand...

Two week cycle
Date:

Foods that need to be consumed on a daily basis:

Food: (P) = Provided by the study<br>(E) = Prepared by the individual	Amount to be consumed daily:	Checklist:
Traditional Cretan bread	8 slices =224g	☐☐☐☐☐☐☐☐
Fruit	3 different varieties of fruit	☐☐☐☐
Greek coffee (P)	Unlimited (at least 1 or more serves per day)	No. of cups per day
Greek mountain tea (P)	Unlimited (at least 1 or more serves per day)	No. of cups per day
Red wine or Beer	220ml of red wine per day or 1 glass of beer	☐

Snack foods to be consumed on a weekly basis:

Type of snack:(P: Provided)		Serves per week:	Checklist:
Milk (whole)		600mls/week	
Chickpeas and sultanas	P	100g of each per week	
Figs (dried)	P	100g per week	
Plain natural yoghurt 1 serve=125g		2 serves/week	☐ ☐
Olives (Black dry and green variety)	P	14 olives/week	
Preserved fruits (2 tablespoons per week only) and Sweet biscuits (1)	P	1 serve each per week	☐ ☐

Sweets/Desserts to be consumed on a monthly basis:

Type of snack:(P: Provided)		Serves per month:	Checklist:
Semolina Sweet	P	1 serve	☐
Milk Pie	P	1 serve	☐
Walnut cake	P	1 serve	☐
Baklava	P	1 serve	☐

Two week cycle
Date:

Types of fruits that can be consumed on a Mediterranean diet

Peach ☐☐☐☐☐☐☐	Mandarin ☐☐☐☐☐	Figs (dried in winter) ☐☐☐	Banana ☐☐☐☐☐☐
Nectarine ☐☐☐☐☐	Green grapes ☐☐☐	Pear ☐☐☐☐☐☐☐	Black grapes ☐☐☐☐☐☐
Orange ☐☐☐☐☐☐	Water melon ☐☐☐☐	Plums ☐☐☐☐☐☐	Other .... ☐☐☐☐
Apple ☐☐☐☐☐☐☐	Rock melon ☐☐☐☐	Pomegranates ☐☐☐☐	Other .... ☐☐☐☐

Breakfast

Please choose only one of the following meal choices for breakfast:

**Meal Choice 1:**<br>Tomato (1 whole tomato) + onion (1/4 of an onion) + oregano + olive oil (1/2 a tablespoon) + bread	☐
**Meal Choice 2:**<br>Sour milk porridge (1/2 a cup) + 1 ½ cups of water + salt + olive oil (1/2 a tablespoon)	☐
**Meal Choice 3:**<br>One boiled egg with one large tomato + olive oil(1/2 a tablespoon) + bread	☐
**Meal Choice 4:**<br>Cucumber (1 whole, medium sized) + olive oil (1/2 a tablespoon) + bread	☐
**Meal Choice 5:**<br>Tomato (1 whole tomato) + onion (1/4 of an onion) + oregano + olive oil (1/2 a tablespoon) + bread	☐
**Meal Choice 6:**<br>Watermelon or rock melon or grapes + bread	☐
**Meal Choice 7:**<br>Bread with honey or jam (all fruit jam) - 1 teaspoon of either honey or jam	☐

Two week cycle
Date:

Lunch

Please choose only one of the following meal choices and salad choices for lunch:

**Meal Choice 1: (P)**<br>Cannelli bean soup (Fassoulada)	☐	**Salad Choice 1: Mixed potato salad (E)** Potato, onion, celery, spring onion, garlic, radish, parsley, salt and olive oil (1 tablespoon)	☐
**Meal Choice 2: (P)**<br>Spinach & rice (Spanakorizo)	☐	**Salad Choice 2: Tomato salad (E)**<br>Tomato, onion, cucumber, lemon juice, salt and olive oil (1 tablespoon)	☐
**Meal Choice 3: (P)**<br>Fish soup (Psarosoupa)	☐	**Salad Choice 3: Lettuce salad (E)**<br>Lettuce, cucumber, spring onion, salt, lemon juice, olive oil (1 tablespoon)	☐
**Meal Choice 4: (E)**<br>1. Asparagus omelette or 2. scrambled eggs with potato	☐	**Salad Choice 4: Cabbage salad (E)**<br>Cabbage , carrot, lemon juice, salt and olive oil (1 tablespoon)	☐
**Meal Choice 5: (P)**<br>Lentil soup (Faki)	☐	**Salad Choice 5: Cauliflower salad (E)**<br>Cauliflower, lemon juice, salt and olive oil(1 tablespoon)	☐
**Meal Choice 6: (E)**<br>Green runner bean casserole with carrot and potato (Fassoulakia)	☐	**Salad Choice 6: Roasted red capsicum (P) + Lettuce salad (E)**<br>lettuce, cucumber, spring onion, salt, lemon juice, olive oil(1/2 a tablespoon)	☐
**Meal Choice 7: (P)**<br>Wrapped vine leaves (Dolmades)	☐	***Salad Choice 7: Wild green salad (P)***<br>*Chicory salad (Radikia)*	☐
**Meal Choice 8: (E)**<br>Okra casserole	☐	***Salad Choice 8: Wild green salad (P)***<br>*Amaranth salad (Vlita)*	☐
**Meal Choice 9: (P)**<br>Chickpea soup (Rivithia)	☐	**Salad Choice 9: Fennel salad (E)**<br>Fennel root with lemon, salt and olive oil (1/2 a tablespoon) + one whole tomato (1/2 a tablespoon)	☐
**Meal Choice 10: (P)**<br>Baked lima beans (Gigantes)	☐	**Salad Choice 10: Broccoli salad (E)**<br>Broccoli, lemon juice, salt and olive oil (1 tablespoon)	☐
**Meal Choice 11: (P)**<br>3 cheese pies (Tiropita)	☐	**Salad Choice 11: Tomato salad (E)**<br>Tomato, onion, cucumber, lemon juice, salt and olive oil (1 tablespoon)	☐
**Meal Choice 12: (P)**<br>3 spinach pies (Spanakopita)	☐	**Salad Choice 12: Cabbage salad (E)**<br>Cabbage, carrot, lemon juice, salt, olive oil (1 tablespoon)	☐
**Meal Choice 13: (E)**<br>Mixed vegetable bake (Briam)	☐	**Salad Choice 13: Runner bean salad (E)**<br>Green runner bean, parsley, garlic, lemon juice, salt and olive oil (1tablespoon)	☐
**Meal Choice 14: (P)**<br>Egg and lemon soup (αυγολεμονο)	☐	**Salad Choice 14: Lettuce salad (E)**<br>Cos lettuce, spring onion, salt, vinegar and olive oil (1 tablespoon)	☐

Date:

Dinner

Please choose only one of the following meal choices and salad choices for dinner:

**Meal Choice 1: (E)** Risoni with lamb (Giouvetsi)	☐	**Salad Choice 1: Mixed potato salad (E)** Potato, onion, celery, spring onion, garlic, radish, parsley, salt and olive oil(1 tablespoon)	☐
**Meal Choice 2: (P)** Cabbage & rice (Lahanoriza)	☐	**Salad Choice 2: Lettuce salad (E)** Lettuce, cucumber, spring onion, salt, lemon juice, olive oil (1 tablespoon)	☐
**Meal Choice 3: (P)** Pasta and mince pie (Pastitsio)	☐	**Salad Choice 3: Cabbage salad (E)** Cabbage , carrot, lemon juice, salt and olive oil (1 tablespoon)	☐
**Meal Choice 4: (E)** Baked fish with roasted potatoes	☐	**Salad Choice 4: Cauliflower salad (E)** Cauliflower, lemon juice, salt and olive oil (1 tablespoon)	☐
**Meal Choice 5: (E)** Baked chicken with roasted potatoes	☐	**Salad Choice 5: Roasted green capsicum (P) + Lettuce salad (E)** Cos lettuce, spring onion, salt, vinegar and olive oil(1/2 a tablespoon)	☐
**Meal Choice 6: (E)** Mixed vegetable bake (Briam)	☐	***Salad Choice 6: Wild green salad (P)*** *Dandelion or sow thistle salad*	☐
**Meal Choice 7: (P)** Stuffed eggplant (Papoutsakia)	☐	**Salad Choice 7: Lettuce and dill salad (E)** Lettuce, dill, vinegar, salt and olive oil (1 tablespoon)	☐
**Meal Choice 8 (P)** Stuffed green capsicum (Gemesta)	☐	**Salad Choice 8: Tomato salad (E)** Tomato, onion, cucumber, lemon juice, salt and olive oil (1/2 a tablespoon)	☐
**Meal Choice 9: (P)** Vegetarian mousaka	☐	**Salad Choice 9: Asparagus salad (E)** Asparagus with lemon juice, salt and olive oil (1 tablespoon)	☐
**Meal Choice 10: (E)** Pork with celery (Fricase)	☐	**Salad Choice 10: Broccoli salad (E)** Broccoli with lemon juice, salt and olive oil (1 tablespoon)	☐
**Meal Choice 11: (E)** Lamb chops with roasted potatoes(Potential Restaurant meal)	☐	***Salad Choice 11: Wild green salad (P)*** *Chicory salad (Radikia)*	☐
**Meal Choice 12: (E)** Bream/Mullets or fried white bait (Potential Restaurant meal)	☐	**Salad Choice 12: Beetroot salad (P) + Lettuce salad (E)** Cos lettuce, spring onion, salt, lemon juice, olive oil (1 tablespoon)	☐
**Meal Choice 13: (E)** Calamari with tomato (Potential Restaurant meal)	☐	**Salad Choice 13: Potato salad (P)** Potato, spring onion, salt, lemon juice, salt and olive oil (1 tablespoon)	☐
**Meal Choice 14: (E)** Pea, carrot and potato casserole (αρακα)	☐	***Salad Choice 14: Wild green salad (P)*** *Endive salad*	☐

Cretan Menu and recipes published in The Mediterranean Diet (Itsiopoulos, C. Pan MacMillan Australia Ltd, Sydney Australia 2013).

Healthy Menu for Chronic Disease Prevention: Diabetes, Heart Disease, Stroke, Dementita

HIGH OMEGA 3 FATS, LOW IN SATURATED FATS, ANTIOXIDANT RICH	BREAKFAST	LUNCH	DINNER	SNACK
SUNDAY	Dakos (Bruchetta)<br>Coffee (Greek/Espresso)<br>Mandarin	Mussels stewed in white wine. Slice grain bread<br>Greek Salad	Eggplant moussaka with lamb mince.<br>Raddichio fennel and walnut salad.	Low Fat Greek yoghurt with berries.<br>Sesame snack bar
MONDAY	Slice grain bread with poached egg and sliced avocado sprinkled with lemon juice and cracked pepper.	Rocket, pear and walnut salad with small tin tuna.<br>Mandarin<br>Plain mineral water	Baked chicken breast, skinless. Boiled broccoli salad. Small baked potato.<br>Beetroot and garlic salad.	Low fat Greek yoghurt with walnuts and honey.<br>Slice watermelon.<br>Greek biscuit (koulourakia)
TUESDAY	Porridge (cooked rolled oats with skim milk) topped with fresh blueberries.<br>Coffee (Greek/Espresso)	Stuffed vine leaves (4-6)<br>Greek coleslaw salad.<br>Eggplant dip	Baked snapper.<br>Salad of boiled greens and beetroot with garlic side salad. Glass white wine	Risogalo dessert<br>Whole orange<br>Almonds (8-10)
WEDNESDAY	Dakos (Bruchetta)<br>Herbal tea<br>Whole orange	Beetroot and runner bean salad with walnuts and feta.<br>Slice grain bread.	Vegetable bake.<br>Greek salad.<br>Mineral water	Dried figs (2-3)<br>Walnuts (30g)<br>Low fat Greek yoghurt with berries
THURSDAY	Porridge (cooked rolled oats with skim milk) topped with fresh blueberries.<br>Herbal tea	Cannellini bean soup.<br>Greek salad<br>Slice grain bread.	Rabbit stew with red wine (can use chicken if prefer)<br>Mixed potato salad<br>Glass red wine	Low fat Greek yoghurt with honey and walnuts.<br>Slice revani cake (or other)<br>1 Apricot
FRIDAY	Slice grain bread with poached egg and sliced avocado.<br>Sprinkled with lemon juice and pepper.	Roasted vegetable open sandwich.<br>Plain mineral water	Baked risoni with lamb.<br>Lettuce, cucumber, spring onion salad.<br>Plain mineral water.	Slice walnut cake.<br>Low fat Greek yoghurt with berries.<br>Slice rock melon.
SATURDAY	Poached eggs in stewed tomatoes.<br>Slice grain bread.<br>Whole orange	Baked sardines on toasted grain bread.<br>Greek coleslaw salad.<br>Plain mineral water.	Stuffed tomatoes with rice.<br>Black eye bean salad.<br>Tzatziki dip<br>Glass white wine.	Baklava (sm. serve).<br>Greek yoghurt with honey.<br>Slice watermelon
8000kj	78 g Protein (16% Energy)	180 g Carbs (38% Energy)	92 g Fat (42% Energy)	6 g Alc (2.2% Energy)
